# Plasma metabolomic profiling of dietary patterns associated with glucose metabolism status: The Maastricht Study

**DOI:** 10.1186/s12916-022-02653-1

**Published:** 2022-11-21

**Authors:** Evan Yi-Wen Yu, Zhewen Ren, Siamak Mehrkanoon, Coen D. A. Stehouwer, Marleen M. J. van Greevenbroek, Simone J. P. M. Eussen, Maurice P. Zeegers, Anke Wesselius

**Affiliations:** 1 Key Laboratory of Environmental Medicine and Engineering of Ministry of Education, Department of Epidemiology & Biostatistics, School of Public Health, Southeast University, Nanjing, 210009 China; 2 Department of Epidemiology, CAPHRI Care and Public Health Research Institute, Maastricht University, Universiteitssingel 40 (Room C5.570), Maastricht, 6229ER The Netherlands; 3 Department of Data Science and Knowledge Engineering, Maastricht University, Maastricht, 6229ER The Netherlands; 4 CARIM School for Cardiovascular Diseases, Maastricht University, Maastricht, 6229ER The Netherlands; 5 Department of Internal Medicine, Maastricht University Medical Center+, Maastricht, 6229HX The Netherlands; 6 School of Nutrition and Translational Research in Metabolism, Maastricht University, Universiteitssingel 40 (Room C5.564), Maastricht, 6229ER The Netherlands

**Keywords:** Dietary patterns, Metabolomics, Glucose metabolism, Cohort study, Molecular epidemiology

## Abstract

**Background:**

Glucose metabolism has been reported to be affected by dietary patterns, while the underlying mechanisms involved remain unclear. This study aimed to investigate the potential mediation role of circulating metabolites in relation to dietary patterns for prediabetes and type 2 diabetes.

**Methods:**

Data was derived from The Maastricht Study that comprised of 3441 participants (mean age of 60 years) with 28% type 2 diabetes patients by design. Dietary patterns were assessed using a validated food frequency questionnaire (FFQ), and the glucose metabolism status (GMS) was defined according to WHO guidelines. Both cross-sectional and prospective analyses were performed for the circulating metabolome to investigate their associations and mediations with responses to dietary patterns and GMS.

**Results:**

Among 226 eligible metabolite measures obtained from targeted metabolomics, 14 were identified to be associated and mediated with three dietary patterns (i.e. Mediterranean Diet (MED), Dietary Approaches to Stop Hypertension Diet (DASH), and Dutch Healthy Diet (DHD)) and overall GMS. Of these, the mediation effects of 5 metabolite measures were consistent for all three dietary patterns and GMS. Based on a 7-year follow-up, a decreased risk for apolipoprotein A1 (APOA1) and docosahexaenoic acid (DHA) (RR 0.60, 95% CI 0.55, 0.65; RR 0.89, 95% CI 0.83, 0.97, respectively) but an increased risk for ratio of ω-6 to ω-3 fatty acids (RR 1.29, 95% CI 1.05, 1.43) of type 2 diabetes were observed from prediabetes, while APOA1 showed a decreased risk of type 2 diabetes from normal glucose metabolism (NGM; RR 0.82, 95% CI 0.75, 0.89).

**Conclusions:**

In summary, this study suggests that adherence to a healthy dietary pattern (i.e. MED, DASH, or DHD) could affect the GMS through circulating metabolites, which provides novel insights into understanding the biological mechanisms of diet on glucose metabolism and leads to facilitating prevention strategy for type 2 diabetes.

**Supplementary Information:**

The online version contains supplementary material available at 10.1186/s12916-022-02653-1.

## Background

Type 2 diabetes contributes enormously to global burdens of mortality and disability, which has been reported to affect around 425 million people worldwide in the past decade with an increased tendency of occurring in adolescent and young adults [1, 2]. In addition, more than 470 million people worldwide are estimated to suffer from prediabetes, a high-risk state of diabetes development, whereof 5% to 10% will progress to type 2 diabetes within a year [3]. Therefore, the identifying of high-risk individuals and the management of glucose metabolism status (GMS) before these conditions manifest is essential.

The aetiology of glucose metabolism disorders (i.e. prediabetes and type 2 diabetes) is multi-factorial, with obesity, physical inactivity and genetic factors being important driving forces [4]. In addition, a large body of research has suggested diet as a key modifiable component in the prevention, development, and management of prediabetes and type 2 diabetes [5, 6]. Many of the previous evidence, however, is based on single food items/ nutrients. Since people do not consume isolated foods/nutrients and a high internal correlation between food items exists, this single food item approach might be in accurate and unable to measure the impact of the interaction among different foods on disease risk. Therefore, a more holistic dietary approach, in which food consumption patterns are analysed, became more popular to capture the complex interaction of nutrients and foods with GMS [7, 8].

A higher adherence to healthy dietary patterns, such as the Mediterranean Diet (MED), Dietary Approaches to Stop Hypertension (DASH) Diet, and Dutch Healthy Diet (DHD) has been reported to be associated with a reduced risk of type 2 diabetes risk and its complications [9–11]. However, due to the complexity of diet composition, an accurate assessment of the specific effect of dietary patterns on human health is still challenging.

In recent years, high-throughput metabolomics techniques have been developed for the quantification of an individual’s comprehensive metabolites profile, making it eligible to objectively measure the dietary biomarkers, which could help to reveal the response to nutritional changes and further identify the early onset of metabolic diseases [12, 13].

Therefore, this study aimed to investigate the mediation role of circulating metabolites in the relation between healthy dietary patterns (i.e. MED, DASH, DHD) and prediabetes and type 2 diabetes, thereby facilitating a more effective nutritional prevention strategy and metabolomic monitoring for glycaemic control.

## Methods

### Study design and population

Data from The Maastricht Study, an observational, prospective, population-based cohort study was used. The rationale and methodology have been described previously [14]. In brief, this study focuses on the aetiology, pathophysiology, complications, and comorbidities of type 2 diabetes and is characterised by an extensive phenotyping approach. Eligible for participation were individuals between 40 and 75 years of age living in the southern part of the Netherlands. Participants were recruited through mass media campaigns and from the municipal registries and the regional Diabetes Patient Registry via mailings. Recruitment was stratified according to known type 2 diabetes status, with an oversampling of individuals with type 2 diabetes for reasons of efficiency. For the current study 3807 participants, who completed the baseline survey between November 2010 and November 2013, were eligible for inclusion. The examinations of each participant were performed within a time window of 3 months after finishing the baseline survey.

**Fig. 1:**
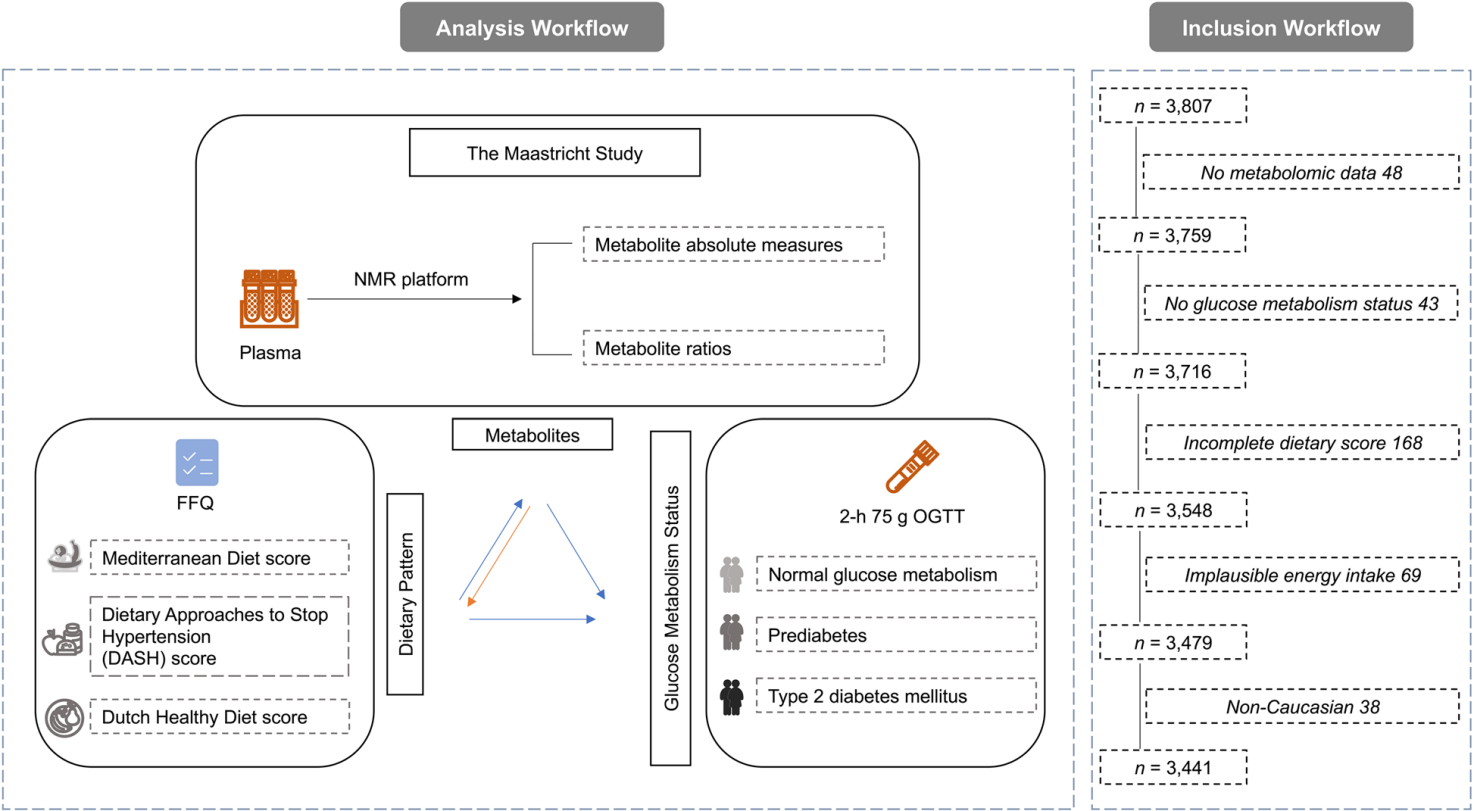
Overview of the study design. Fasting blood samples were obtained, and plasma were extracted to quantify the circulation metabolites based on NMR platform. Diet was assessed at baseline by a validated, self-administered FFQ developed based on the Dutch national FFQ tool, which was then calculated into dietary patterns, i.e. Mediterranean diet (MED), Dietary Approaches to Stop Hypertension (DASH) Diet, and Dutch Healthy Diet (DHD). Participants underwent a standardized 2-h 75-g oral glucose tolerance test (OGTT) after fasting overnight along with information about diabetes medication to determine the glucose metabolism status (GMS), which was defined based on the World Health Organization 2006 criteria as; normal glucose metabolism, NGM, fasting plasma glucose <6.1 mmol/L; prediabetes, fasting plasma glucose of 6.1–6.9 mmol/L and no hypoglycaemic medications; and type 2 diabetes, fasting plasma glucose ≥7.0 mmol/L or hypoglycaemic medications. For safety reasons, participants using insulin or with a fasting plasma glucose (FPG) value above 11.0 mmol/L (determined by finger prick) did not undergo the OGTT. For these individuals, the FPG value and diabetes medication information was used to determine GMS. The exclusion criteria for participants included in the further analysis were performed as follows: (1) 48 participants without measured metabolites; (2) 43 participants without information on glucose metabolism status; (3) 168 participants with implausible energy intakes (<800 or >4200 kcal/day for men, and <500 or >3500 kcal/day for women); (4) 69 participants of whom data on dietary assessment were incomplete for the calculation of dietary patterns; (5) 38 non-Caucasian participants. Abbreviations: NMR, magnetic resonance spectroscopy; FFQ, food frequency questionnaire; MED, Mediterranean diet; DASH, Dietary Approaches to Stop Hypertension; DHD, Dutch Healthy Diet; OGTT, oral glucose tolerance test; GMS, glucose metabolism status; NGM, normal glucose metabolism; FPG, fasting plasma glucose

The exclusion criteria for participants included in the further analysis were performed as follows: (1) 48 participants without measured metabolites; (2) 43 participants without information on glucose metabolism status; (3) 168 participants with implausible energy intakes (<800 or >4200 kcal/day for men, and <500 or >3,500 kcal/day for women) [15]; (4) 69 participants of whom data on dietary assessment was incomplete for the calculation of dietary patterns; (5) 38 non-Caucasian participants. Finally, a total of 3441 participants, of which 1960 individuals with normal glucose metabolism (NGM), 514 with prediabetes and 967 with type 2 diabetes by design, were included in the current study (Fig. 1).

### Metabolomics quantification and processing

Fasting blood samples were collected in EDTA (ethylenediaminetetraacetic acid) tubes. After centrifuging, plasma was stored at −80°C. Metabolite measures were quantified from EDTA plasma samples using the high-throughput 1H-NMR (nuclear magnetic resonance) metabolomics platform (Nightingale Health Ltd., Helsinki, Finland; https://nightingalehealth.com) at two separate occasions, i.e. years 2014 and 2016, respectively [16]. This platform provides simultaneous quantification of individual metabolites and metabolite ratios, details of the experimentation and applications of the NMR metabolomics platform have been described previously [17].

Metabolite measures that failed quality control (i.e. glutamine, pyruvate, glycerol, β-hydroxybutyrate, and acetate) were excluded from the analysis. The final set of metabolite measures included 145 metabolites and 81 metabolite ratios in the current study, comprising total lipid concentrations, fatty acids composition and low-molecular-weight metabolites including ketone bodies, glycolysis-related metabolites, amino-acids, and metabolites related to immunity and fluid balance. To enhance interpretation, metabolite measures were classified into 3 clusters curated by Nightingale Health [17]: (1) lipid composition and particle concentration for lipoprotein subclasses (*n*=98); (2) lipids, fatty acids and various low-molecular-weight metabolites (*n*=47); and (3) metabolite ratios (*n*=81) (Additional file 1: Table S1).

Missing data for absolute metabolite measures were imputed using R package *Missforest* [18]. Zero values in absolute measures were replaced by half of the lowest value of each corresponding metabolite. Then, metabolite ratios were recalculated according to the complete matrix of metabolites. In order to normalize all the metabolite measures, a 10th percentile value (based on the distribution of the absolute measure/ratio under investigation) was added and subsequently ln-transformed. Finally, the obtained values were scaled to standard deviation units with normal distributions.

### Assessment of glucose metabolism status

Participants underwent a standardized 2-h 75-g oral glucose tolerance test (OGTT) after fasting overnight along with information about diabetes medication to determine the glucose metabolism status (GMS), which was defined based on the World Health Organization 2006 criteria as; normal glucose metabolism, NGM, fasting plasma glucose <6.1 mmol/L (GMS score=0); prediabetes, fasting plasma glucose of 6.1–6.9 mmol/L and no hypoglycaemic medications (GMS score=1); and type 2 diabetes, fasting plasma glucose ≥7.0 mmol/L or hypoglycaemic medications (GMS score=2) [19]. For safety reasons, participants using insulin or with a fasting plasma glucose (FPG) value above 11.0 mmol/L (determined by finger prick) did not undergo the OGTT. For these individuals, the FPG value and diabetes medication information were used to determine GMS. A higher GMS score indicates a poorer glycaemic control, and each pair of GMS comparison was defined as that the GMS was developed from NGM to prediabetes, from NGM to type 2 diabetes, or from prediabetes to type 2 diabetes.

### Assessment of dietary patterns

Diet was assessed at baseline by a validated, self-administered food frequency questionnaire (FFQ), i.e. Maastricht-FFQ, developed based on the Dutch national FFQ tool [20]. The Maastricht-FFQ assessed the amount and frequency of intakes from 23 product groups comprising 253 food items within a reference period of one year. Intake of total energy and nutrients was calculated using the Dutch Food Composition Database (NEVO) [21]. More details on the development and the validity of this FFQ have been reported elsewhere [22]; briefly, the Maastricht-FFQ selected food items with the largest contributions to both absolute intake and explained variance in intake of energy and 24 nutrients based on the Dutch National Food Consumption Survey 2007–2010 [23]. In addition, the Maastricht-FFQ was validated within the Nutrition Questionnaires plus (NQplus) study population [24], where the population characteristics in The Maastricht Study [25] were generally comparable with the NQplus population with respect to age, BMI, smoking status, educational attainment and food items.

#### Mediterranean Diet (MED) score

The MED score was based on 9 food components (i.e. vegetables, legumes, fruits and nuts, fish, cereals, dairy, meat, ratio [MUFA (monounsaturated fatty acids) + PUFA (polyunsaturated fatty acids)]/SFA (saturated fatty acids), and alcohol, with sex-specific medians of intakes as cut-off values. The median intake for food groups was derived from the FFQ. For healthy components (i.e. vegetables, legumes, fruits and nuts, fish, cereals, ratio (MUFA+PUFA)/SFA), a score of 0 was assigned for intake below the median of each food component, while a score of 1 was granted for intake higher or equal to the median of each food component. For unhealthy food components (i.e. red and processed meats, and dairy products), the scores were inverted (1 for intake below the median, 0 for intake above the median). Regarding alcohol consumption, a score of 1 was allocated if consumption was between 10 and 50 g/day for males and between 5 and 25 g/day for females, and a score of 0 for any other amount of alcohol consumption. The sum of the scores for each food component resulted in the overall MED score (minimum 0, maximum 9) [26].

#### Dietary Approaches to Stop Hypertension (DASH) score

The DASH score was based on 8 food components (i.e. vegetables, fruits, nuts and legumes, wholegrain products, low-fat dairy, red and processed meat, sugar sweetened beverages, and sodium intake) with sex-specific quintiles as cut-off values. For each food component, the score ranged between 1 and 5, which was assigned proportionally to the intake level. A higher intake of healthy food components (i.e. vegetables, fruits, nuts and legumes, wholegrain products, low-fat dairy) or unhealthy food components (i.e. red and processed meat, sugar-sweetened beverages, and sodium intake) corresponded to higher scores. The sum of the scores for each food group resulted in the overall DASH score (minimum 8, maximum 40) [27].

#### Dutch Healthy Diet (DHD) score

The DHD score (version 2015) was used to measure the adherence to Dutch dietary guidelines of 2015, consisting of 15 food components (i.e. vegetables, fruits, wholegrain products, legumes, nuts, fish, tea, dairy, fats and oils, coffee, red meat, processed meat, sweetened beverages and fruit juices, alcohol, and salt) [28]. For each healthy food component (i.e. vegetables, fruits, wholegrain products, legumes, nuts, fish, tea), intake equal to or higher than a cut-off value, specified according to the dietary guidelines, the maximum score (score=10) was given, while for intakes below that cut-off value the score was calculated by means of linear interpolation between threshold value (score=0) and cut-off value (score=10). For each unhealthy food component (i.e. dairy, fats and oils, red meat, processed meat, sweetened beverages and fruit juices, alcohol, and salt), the maximum score (score=10) was assigned if the intake was equal to or below a specific cut-off value, whereas for intake higher than that cut-off value the score was calculated by means of linear interpolation between the cut-off value (score=10) and the threshold value (score=0). The sum of the scores for each food group resulted in the overall DHD score (minimum 0, maximum 140). A detailed description of the operationalization has been described elsewhere [28] (Additional file 1: Table S2).

### Assessment of covariates

Covariates that were extracted from the questionnaire included age (years, continuous), sex (male or female), body mass index (BMI, kg/m^2^, continuous), education level (low, middle, or high), household income (<2000 euros/month, 2000–3750 euros/month, or ≥3750 euros/month), smoking status (never, current, or former smoker), energy intake (kcal/day, continuous), daily glucose intake (mmol/mol, continuous), estimated glomerular filtration rate (eGFR, ml/min, continuous), total physical activity (h/week, continuous) [29], history of cardiovascular disease (yes or no), use of lipid-modification medication (yes or no), and the year for metabolomics measurement (2014 or 2016). Covariates were obtained from physical examination, laboratory assessment, FFQ and medication interview. For categorial variables, missing data, i.e. smoking status (1.28%), level of education (1.95%), level of household income (4.02%), and history of cardiovascular disease (1.66%), was replaced by an indicator (using 0 as unknown); for continuous variables, missing data, i.e. total physical activity (7.46%), was replaced by the mean value of the total physical activity separated for sexes.

### Statistical analysis

Descriptive statistics are presented as mean [±SD (standard deviation)] or median (interquartile range) for continuous variables, and frequency (percentage, %) for categorial variables. Differences between NGM, prediabetes and type 2 diabetes at baseline, including 3 dietary-pattern scores, were compared by ANOVA test for continuous variables, and chi-squared test for categorial variables.

The dietary pattern scores were divided into 3 tertiles for standardization: low adherence (tertile 1), middle adherence (tertile 2), and high adherence (tertile 3). Firstly, we performed a crude and adjusted ordinal logistic regression analysis (STATA package *ologit* [30]) to evaluate the association between each dietary pattern and the GMS score (i.e. NGM vs. prediabetes vs. type 2 diabetes). In addition, a logistic regression analysis was performed for the compare the GMS score in pairs (i.e. pair 1; GMS score 0 (NGM) vs 1 (prediabetes), pair 2 ; GMS score 0 (NGM) vs 2 (type 2 diabetes), pair 3; GMS score 1 (prediabetes) vs 2 (type 2 diabetes)), based on a crude and adjusted model (STATA package *logit* [31]). The main interaction terms (GMS score with sex and BMI) were added to the adjusted model (*P*-interaction<0.05 was considered statistically significant).

To further investigate the associations of dietary patterns with glucose metabolism, i.e. Homeostatic Model Assessment for Insulin Resistance (HOMA-IR) and haemoglobin A1c (HbA1c), we employed a linear regression analysis with stratification on sexes and BMI (i.e. obesity: BMI≥30 kg/m^2^, and non-obesity: BMI<30 kg/m^2^). For all analyses, lowest tertile of adherence was used as the reference group. A *P* value for trend test was conducted by assigning medians to per tertile as a continuous variable in the models.

Secondly, we used a linear regression analysis (STATA package *regression* [32]) to examine the associations between each dietary pattern and metabolite measures. For this, each metabolite measure was used as the dependent variable, and dietary pattern scores were used as the independent variable. In addition, the metabolite measures associated with the GMS score and with the defined GMS pairs were assessed based on ordinal and binary logistic regression respectively. Again, both crude and adjusted models were performed.

Thirdly, an adjusted mediation analysis was used to examine whether the metabolite measures that were both associated with dietary patterns and GMS are potential mediators. The significance of the mediated effects was assessed using Sobel-Goodman mediation Test (STATA package *sgmediation* [33]).

Finally, to assess the relative risk (RR) of type 2 diabetes developed from NGM or prediabetes according to the identified metabolite measures, a Poisson regression analysis was performed using self-report incidences of type 2 diabetes obtained from a yearly follow-up till 7 years (mean=5.83, standard deviation=0.91).

All the adjusted models mentioned above were performed with the adjustments of continuous covariates, i.e. age (years), BMI ((kg/m^2^), energy intake (kcal/day), daily glucose intake (mmol/mol), eGFR (ml/min), total physical activity (h/week); and categorial covariates, sex (male or female), education level (low, middle, or high), household income (<2,000 euros/month, 2000–3750 euros/month, or ≥3750 euros/month), smoking status (never, current, or former), history of cardiovascular disease (yes or no), use of lipid-modification medication (yes or no), and the year for metabolomics measurement (2014 or 2016) if applicable.

Sensitivity analyses were performed, for the identified metabolites by excluding the participants with incomplete data on covariates and newly type 2 diabetes diagnosed at baseline. All statistical tests were two sided. We calculated the false discovery rate (FDR) to correct for multiple testing at α<0.05 significance level.

## Results

### Characteristics of the study participants and adherence to the dietary patterns

In total 3441 individuals were included in the current study with a mean age of 60 years and 49% (1689) women. Of all included participants, 1960 (57%) and 514 (15%) participants were shown to be NGM and prediabetes respectively, while 967 (28%) participants were diagnosed with type 2 diabetes including 135 type 2 diabetes cases diagnosed at baseline. All baseline characteristics were found to be different across the GMS at *P*<0.05 (Table 1). In addition, the participants with type 2 diabetes tended to be older (63 years), less physically active (11.0 h/week), smokers (70%) and have a lower education level (45%), lower income level (33%), and higher BMI (29.90 kg/m^2^) to individuals with NGM and prediabetes (*P*<0.001). The means of dietary-pattern scores were shown to be lower in NGM and prediabetes compared to type 2 diabetes (*P*<0.001) (Table 1). In addition, the statistical interaction was observed between all dietary patterns and BMI for the GMS score, while only MED showed borderline statistical interaction with sex (Table 2).

**Table 1:** Characteristics of participants with different glucose metabolism status in The Maastricht Study^a^

Characteristics	Total (***n***=3441)	Glucose metabolism status			
		NGM (***n***=1960)	Prediabetes (***n***=514)	Type 2 diabetes (***n***=967)	***P***^†^
Women (%)	1689 (49.08)	1135 (57.91)	242 (47.08)	312 (32.26)	<0.001
Age^b^, years (mean ± SD)	60.14 ± 8.21	58.32 ± 8.14	61.96 ± 7.66	62.86 ± 7.68	<0.001
Education (%)					<0.001
Low	1144 (33.25)	525 (26.79)	181 (35.21)	438 (45.29)	
Middle	929 (27.00)	525 (26.79)	145 (28.21)	259 (26.78)	
High	1301 (37.81)	882 (45.00)	174 (33.85)	245 (25.34)	
Unknown	67 (1.95)	28 (1.43)	14 (2.72)	25 (2.59)	
Smoking status (%)					<0.001
Never	1190 (34.58)	771 (39.34)	151 (29.38)	268 (27.71)	
Former	1774 (51.55)	947 (48.32)	297 (57.78)	530 (54.81)	
Current	433 (12.58)	228 (11.63)	60 (11.67)	145 (14.99)	
Unknown	44 (1.28)	14 (0.72)	6 (1.17)	24 (2.48)	
Household Income (%)					<0.001
Low	1012 (29.41)	537 (27.40)	154 (29.96)	321 (33.20)	
Middle	1384 (40.22)	784 (40.00)	217 (42.22)	383 (39.61)	
High	769 (22.35)	533 (27.19)	533 (28.75)	134 (13.86)	
Unknown	276 (8.02)	106 (5.41)	41 (7.98)	129 (13.34)	
History of CVD (%)	595 (17.29)	249 (12.70)	78 (15.18)	268 (27.71)	
Physical activity (h/week)	12.75 (8.25, 18.25)	13.75 (9.00, 19.25)	13.13 (8.00, 18.25)	11.00 (6.75, 16.75)	<0.001
eGFR, ml min−1 1.73 m−2 (mean ± SD)	79.97 ± 15.65	79.65 ± 14.14	78.88 ± 14.42	81.19 ± 18.82	<0.001
HbA1c (% mmol/mol)	5.6 (5.4, 6.2)	5.4 (5.2, 5.6)	5.7 (5.4, 6.0)	6.6 (6.3, 7.2)	<0.001
Total glucose intake, mmol/L (mean ± SD)	15.21 (10.84, 20.24)	15.79 (11.40, 21.04)	14.88 (10.64, 19.82)	13.89 (9.88, 18.70)	<0.001
BMI, kg/m^2^ (mean ± SD)	27.06 ± 4.53	25.52 ± 3.57	27.71 ± 4.28	29.90 ± 4.97	<0.001
Lipid-modifying medication (yes)	1,222 (36.00)	337 (17.34)	183 (36.02)	702 (74.52)	<0.001
Daily energy intake, kcal/day (mean ± SD)	2,172.22 ± 603.17	2,176.84 ± 599.66	2195.33 ± 592.22	2146.60 ± 615.93	0.030
Fasting glucose, mmol/L (mean ± SD)	6.04 ± 1.62	5.18 ± 0.42	5.90 ± 0.59	7.89 ± 2.00	<0.001
MED score (range 0–9)	4.52 ± 1.64	4.67 ± 1.67	4.55 ± 1.63	4.22 ± 1.54	<0.001
DASH score (range 8–40)	23.97 ± 4.57	24.30 ± 4.63	23.83 ± 4.57	23.36 ± 4.36	<0.001
DHD score (range 0–140)	83.36 ± 14.73	85.12 ± 14.54	82.50 ± 15.11	79.99 ± 14.42	<0.001

The intervals of glucose metabolism statuses were defined as follows: NGM, fasting plasma glucose <6.1 mmol/L; prediabetes, fasting plasma glucose of 6.1–6.9 mmol/L and no hypoglycaemic medications; type 2 diabetes, fasting plasma glucose ≥7.0 mmol/L or hypoglycaemic medications

*P*<0.05 was considered statistically significant

*Abbreviations*: *NGM* normal glucose status, *SD* standard deviation, *BMI* body mass index, *kcal* kilocalories, *MED* Mediterranean Diet, *DASH* Dietary Approaches to Stop Hypertension diet, *DHD* Dutch Healthy Diet

^†^*P* values were calculated by the ANOVA test for continuous variables, or chi*-*squared test for categorial variables

^a^Baseline characteristics were expressed with as mean ± standard deviation, median (interquartile range) or *n* (%)

^b^Age at the time of recruitment

**Table 2:** Associations of dietary patterns with risk of glucose metabolism status in The Maastricht Study

Variables	Tertile groups	***N***	Crude model		Adjusted model	
			OR	95% CI	OR	95% CI
***Overall GMS***						
MED	Tertile 1 (0–3)	1678	Ref.		Ref.	
	Tertile 2 (4–5)	809	0.91	0.77, 1.07	0.87	0.73, 1.05
	Tertile 3 (6–9)	954	0.66	0.56, 0.77	0.59	0.50, 0.70
	*P* for trend		<0.001		0.008	
	*P* for interaction with sex				0.067	
	*P* for interaction with BMI				0.042	
DASH	Tertile 1 (9–22)	1312	Ref.		Ref.	
	Tertile 2 (23–26)	1117	0.83	0.71, 0.98	0.77	0.65, 0.92
	Tertile 3 (26–38)	1012	0.69	0.58, 0.81	0.58	0.48, 0.69
	*P* for trend		<0.001		0.001	
	*P* for interaction with sex				0.052	
	*P* for interaction with BMI				0.037	
DHD	Tertile 1 (32–77)	1147	Ref.		Ref.	
	Tertile 2 (78–89)	1147	0.77	0.64, 0.93	0.79	0.63, 0.98
	Tertile 3 (90–130)	1147	0.52	0.42, 0.63	0.69	0.55, 0.87
	*P* for trend		0.001		<0.001	
	*P* for interaction with sex				0.049	
	*P* for interaction with BMI				0.026	
***NGM vs. prediabetes***						
MED	Tertile 1 (0–3)	1142	Ref.		Ref.	
	Tertile 2 (4–5)	579	1.03	0.81, 1.31	1.01	0.78, 1.28
	Tertile 3 (6–9)	753	0.88	0.70, 1.11	0.79	0.61, 0.97
	*P* for trend		0.314		0.459	
DASH	Tertile 1 (9–22)	896	Ref.		Ref.	
	Tertile 2 (23–26)	802	0.79	0.62, 1.01	0.79	0.62, 1.00
	Tertile 3 (26–38)	776	0.67	0.53, 0.86	0.68	0.53, 0.86
	*P* for trend		0.041		0.036	
DHD	Tertile 1 (32–77)	747	Ref.		Ref.	
	Tertile 2 (78–89)	810	0.82	0.65, 1.04	0.74	0.58, 0.95
	Tertile 3 (90–130)	917	0.65	0.51, 0.83	0.59	0.45, 0.76
	*P* for trend		0.001		0.003	
***NGM vs. type 2 diabetes***						
MED	Tertile 1 (0–3)	1505	Ref.		Ref.	
	Tertile 2 (4–5)	729	0.86	0.71, 1.05	0.98	0.88, 1.18
	Tertile 3 (6–9)	854	0.51	0.42, 0.62	0.61	0.49, 0.77
	*P* for trend		<0.001		<0.001	
DASH	Tertile 1 (9–22)	1109	Ref.		Ref.	
	Tertile 2 (23–26)	955	0.77	0.64, 0.93	0.79	0.63, 0.98
	Tertile 3 (26–38)	863	0.52	0.42, 0.63	0.69	0.55, 0.87
	*P* for trend		<0.001		<0.001	
DHD	Tertile 1 (32–77)	964	Ref.		Ref.	
	Tertile 2 (78–89)	976	0.74	0.62, 0.89	0.85	0.68, 1.06
	Tertile 3 (90–130)	987	0.43	0.35, 0.52	0.62	0.48, 0.78
	*P* for trend		<0.001		<0.001	
***Prediabetes vs. type 2 diabetes***						
MED	Tertile 1 (0–3)	779	Ref.		Ref.	
	Tertile 2 (4–5)	356	0.83	0.63, 1.08	0.81	0.62, 1.06
	Tertile 3 (6–9)	346	0.63	0.48, 0.82	0.60	0.46, 0.78
	*P* for trend		<0.001		0.005	
DASH	Tertile 1 (9–22)	619	Ref.		Ref.	
	Tertile 2 (23–26)	477	0.95	0.74, 1.22	0.93	0.72, 1.20
	Tertile 3 (26–38)	385	0.77	0.59, 1.01	0.73	0.56, 0.96
	*P* for trend		0.026		0.037	
DHD	Tertile 1 (32–77)	583	Ref.		Ref.	
	Tertile 2 (78–89)	508	0.90	0.70, 1.16	0.88	0.68, 1.13
	Tertile 3 (90–130)	390	0.66	0.50, 0.86	0.64	0.49, 0.83
	*P* for trend		0.002		0.001	

The intervals of glucose metabolism statuses were defined as follows: NGM, fasting plasma glucose <6.1 mmol/L; prediabetes, fasting plasma glucose of 6.1–6.9 mmol/L and no hypoglycaemic medications; type 2 diabetes, fasting plasma glucose ≥7.0 mmol/L or hypoglycaemic medications

The adjustments included age (years, continuous), sex (male or female), BMI (kg/m2, continuous), level of education (low, middle or high), level of household income (<2000 euros/month, 2000–3750 euros/month, or ≥3750 euros/month), smoking status (never, current or former smoker), daily energy intake (kcal/day, continuous), daily glucose intake (mmol/mol, continuous), estimated glomerular filtration rate (eGFR, ml/min, continuous), total physical activity (h/week, continuous), usage of lipid-modification medication (no or yes), history of cardiovascular disease (no or yes), and the year for metabolomics measurement (2014 or 2016) if applicable

Reference group was tertile 1

*P*<0.05 was considered statistically significant

*Abbreviations*: *NGM* normal glucose metabolism, *BMI* body mass index, *kcal* kilocalories, *MED* Mediterranean Diet, *DASH* Dietary Approaches to Stop Hypertension diet, *DHD* Dutch Healthy Diet

### Association between dietary patterns and glucose metabolism status

Higher adherence to a healthy dietary pattern was associated with lower odds of an increased GMS score (highest tertile vs lowest tertile: OR-MED_adjust_ 0.59, 95% CI 0.50, 0.70; OR-DASH_adjust_ 0.58, 95% CI 0.48, 0.69; OR-DHD_adjust_ 0.69, 95% CI 0.55, 0.87). Comparing NGM vs. prediabetes, NGM vs. type 2 diabetes, and prediabetes vs. type 2 diabetes, high adherence to all dietary patterns showed a lower odds of developing either prediabetes or type 2 diabetes (Table 2). In addition, higher adherence to a healthy dietary pattern showed to be negatively associated with HOMA-IR (stdβ (95% CI): −0.086 (−0.111, −0.061), −0.034 (−0.043, −0.024), −0.013 (−0.016, −0.010) for MED, DASH and DHD, respectively) and HbA1c (−0.716 (−0.913, −0.519), −0.191 (−0.262, −0.121), −0.098 (−0.120, −0.076) for MED, DASH, and DHD, respectively). Moreover, higher adherence to a healthy dietary pattern showed to be negatively associated with HbA1c in both non-obesity (BMI<30 kg/m^2^) and obesity (BMI≥30 kg/m^2^) participants, while higher adherence to a healthy dietary pattern was negatively associated with HOMA-IR in obesity participants but not in non-obesity participants (Table 3).

**Table 3:** Associations of dietary patterns with HOMA-IR and HbA1c in The Maastricht Study

Variables	Overall			Male			Female			Obesity (BMI≥30)			Non-obesity (BMI<30)		
	*β*	95% CI	*P*	*β*	95% CI	*P*	*β*	95% CI	*P*	*β*	95% CI	*P*	*β*	95% CI	*P*
***HOMA-IR***															
MED	−0.086	−0.111, −0.061	<0.001	−0.094	−0.132, −0.056	<0.001	−0.082	−0.114, −0.051	<0.001	−0.058	−0.130, 0.014	0.114	−0.056	−0.077, −0.036	<0.001
DASH	−0.034	−0.043, −0.024	<0.001	−0.136	−0.213, −0.058	<0.001	−0.029	−0.043, −0.015	<0.001	−0.030	−0.057, 0.004	0.065	−0.018	−0.026, −0.011	<0.001
DHD	−0.013	−0.016, −0.010	<0.001	−0.009	−0.013, −0.006	<0.001	−0.011	−0.015, −0.007	<0.001	−0.009	−0.017, 0.001	0.076	−0.007	−0.010, −0.005	<0.001
***HbA1c***															
MED	−0.716	−0.913, −0.519	<0.001	−0.404	−0.636, −0.173	<0.001	−1.075	−1.382, −0.766	<0.001	−1.052	−1.586, −0.519	<0.001	−0.415	−0.603, −0.226	<0.001
DASH	−0.191	−0.262, −0.121	<0.001	−0.187	−0.269, −0.104	<0.001	−0.187	−0.269, −0.104	<0.001	−0.335	−0.531, −0.139	<0.001	−0.061	−0.129, −0.006	0.007
DHD	−0.098	−0.120, −0.076	<0.001	−0.049	−0.076, −0.021	<0.001	−0.092	−0.128, −0.057	<0.001	−0.148	−0.207, −0.089	<0.001	−0.044	−0.065, −0.023	<0.001

The intervals of glucose metabolism statuses were defined as follows: NGM, fasting plasma glucose <6.1 mmol/L; prediabetes, fasting plasma glucose of 6.1–6.9 mmol/L and no hypoglycaemic medications; type 2 diabetes, fasting plasma glucose ≥7.0 mmol/L or hypoglycaemic medications

The adjustments included age (years, continuous), sex (male or female), BMI (kg/m^2^, continuous), level of education (low, middle or high), level of household income (<2000 euros/month, 2000–3750 euros/month, or ≥3750 euros/month), smoking status (never, current or former smoker), daily energy intake (kcal/d, continuous), daily glucose intake (mmol/mol, continuous), estimated glomerular filtration rate (eGFR, ml/min, continuous), total physical activity (h/week, continuous), usage of lipid-modification medication (no or yes), history of cardiovascular disease (no or yes), and the year for metabolomics measurement (2014 or 2016) if applicable

Reference group was tertile 1

*P*<0.05 was considered statistically significant

*Abbreviations*: *NGM* normal glucose metabolism, *BMI* body mass index, *kcal* kilocalories, *MED* Mediterranean Diet, *DASH* Dietary Approaches to Stop Hypertension diet, *DHD* Dutch Healthy Diet, *HOMA-IR* homeostatic model assessment for insulin resistance, *HbA1c* haemoglobin A1c

### Variation of the plasma metabolome in response to the dietary patterns

Of the 226 metabolite measures (i.e. 145 metabolites and 81 metabolite ratios) used in the analyses, we identified 33 (14.60%; stdβ ranged −0.071 to 0.091), 59 (26.11%; stdβ ranged −0.018 to 0.020), and 64 (28.32%; stdβ ranged −0.010 to 0.012) metabolite measures significantly associated with adherence to the MED, DASH, and DHD score, respectively (*P*_-FDR_<0.05). Of these, 17 metabolite measures were shown to be associated with all three dietary patterns, whereof the associations of 14 metabolite measures were positive and the other associations of 3 metabolite measures (isoleucine, ratio of phospholipids to total lipids in medium LDL (%), ratio of triglycerides to total lipids in medium HDL (%)) were negative. These metabolite measures were classified as cholesterol (*n*=1), apolipoproteins (*n*=1), branched-chain amino acids (BCAA; *n*=1), fatty acids (*n*=3), and metabolite ratios (*n*=11) (Additional file 1: Table S4).

### Associations of metabolite measures with different glucose metabolism status

Seventy-eight (35%) metabolite measures were identified to be associated with an increased GMS score as well as with the different GMS pairs at FDR<0.05, whereof the associations of 34 metabolite measures were positive and the other associations of 44 metabolite measures were negative (Additional file 1: Table S4). When comparing the 17 dietary-pattern-associated metabolite measures and the 78 GMS-associated metabolite measures, we found 14 metabolite measures were significantly associated with both dietary patterns and GMS, including 6 metabolites and 8 metabolite ratios. Of these, associations of 12 metabolite measures were shown to be positively associated with dietary patterns but negatively associated with an increased GMS score, while 2 metabolite measures, i.e. isoleucine and ratio of ω-6 to ω-3 fatty acids, were negatively associated with dietary patterns but positively associated with an increased GMS score (Additional file 1: Tables S3 and S4). In addition, all the 14 metabolite measures identified above showed to be associated with levels of HOMA-IR and HbA1c; again, higher isoleucine (stdβ 0.182, *P*_-FDR_ 1.50×10^−15^; stdβ 0.895, *P*_-FDR_ 7.03×10^−8^, respectively) and the ratio of ω-6 to ω-3 fatty acids (stdβ 0.130, *P*_-FDR_ 1.42×10^−26^; stdβ 0.547, *P*_-FDR_ 1.48×10^−4^, respectively) showed to be associated with higher HOMA-IR and HbA1c levels, while other metabolites with lower levels (Additional file 1: Table S4).

### Mediation of metabolite measures in the relationship between dietary patterns and glucose metabolism status

**Fig. 2:**
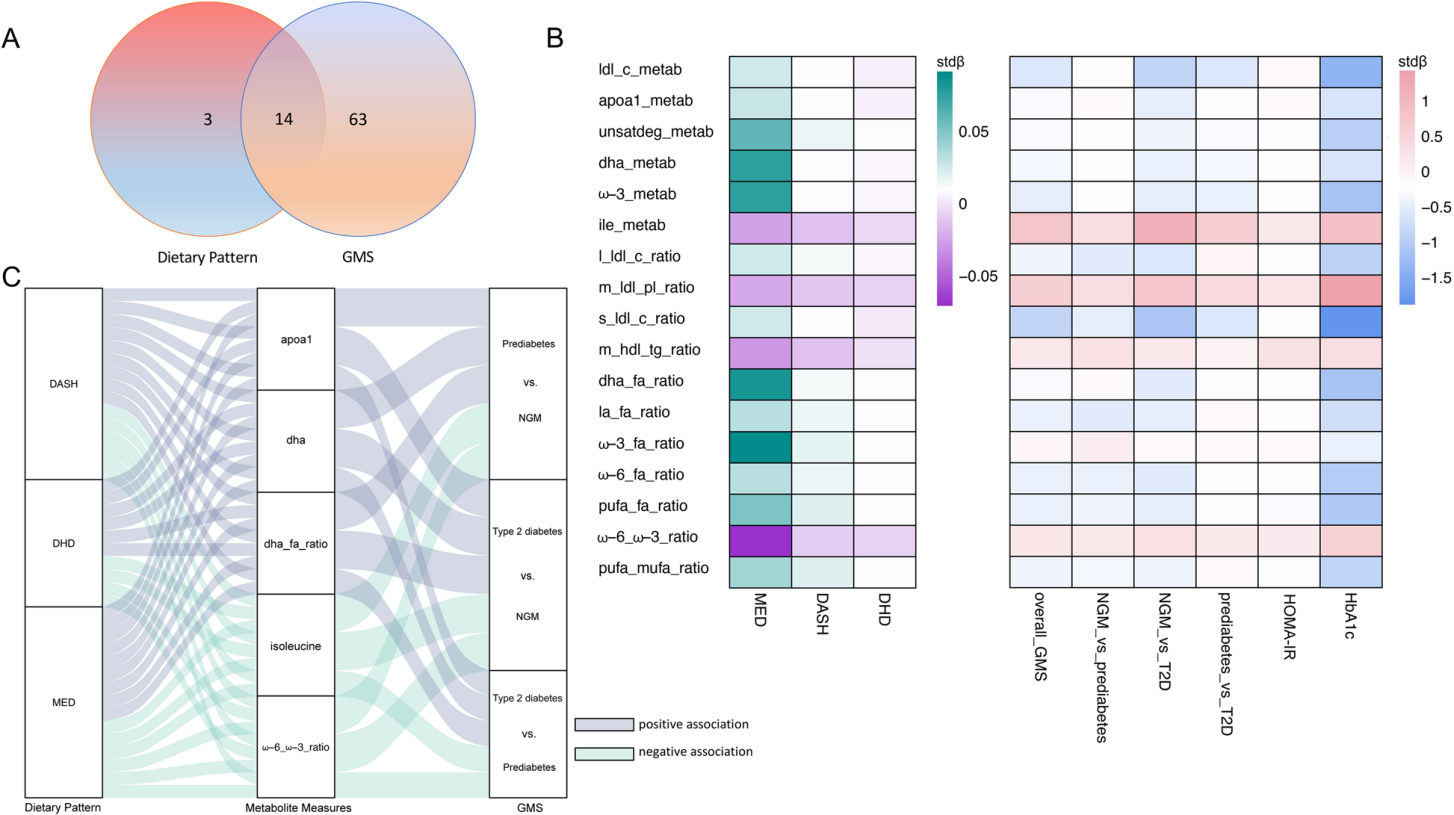
Metabolite measures associated and mediated between dietary patterns and glucose metabolism status. **A** Plasma metabolite measures associated with three dietary patterns and glucose metabolism status with their overlap. **B** Associations of metabolite measures with dietary patterns, glucose metabolism status, HOMA-IR and HbA1c. Red/green squares indicate positive associations, while blue/purple squares indicate negative associations. **C** Parallel coordinates chart showing the 14 significant mediated effects of plasma metabolite measures. The left panel shows the dietary patterns, the middle panel shows the plasma metabolite measures, and the right panel shows the pairs of glucose metabolism status. The curved lines across panels indicate the mediated effects, while the colours correspond to different associations (i.e. grey for positive/negative, and green for negative/positive). Abbreviations: MED, Mediterranean Diet; DASH, Dietary Approaches to Stop Hypertension diet; DHD, Dutch Healthy Diet; GMS, glucose metabolism status; NGM, normal glucose metabolism; T2D, type 2 diabetes; HOMA-IR, Homeostatic Model Assessment for Insulin Resistance; HbA1c, haemoglobin A1c; ldl_c_metab, total cholesterol in very small VLDL (mmol/l); apoa1_metab, apolipoprotein A-I (g/l); unsatdeg_metab, estimated degree of unsaturation; dha_metab, 22:6, docosahexaenoic acid (mmol/l); ω-3_metab, ω-3 fatty acids (mmol/l); ile_metab, isoleucine; l_ldl_c_ratio, total cholesterol to total lipids ratio in large LDL (%); m_ldl_c_ratio, total cholesterol to total lipids ratio in medium LDL (%); s_ldl_c_ratio, total cholesterol to total lipids ratio in small LDL (%); m_hdl_tg_ratio, triglycerides to total lipids ratio in medium HDL (%); dha_fa_ratio,ratio of 22:6 docosahexaenoic acid to total fatty acids (%); la_fa_ratio, ratio of 18:2 linoleic acid to total fatty acids; ω-3_fa_ratio, ratio of ω-3 fatty acids to total fatty acids (%); ω-6_fa_ratio, ratio of ω-6 fatty acids to total fatty acids (%); pufa_fa_ratio, ratio of polyunsaturated fatty acids to total fatty acids (%); ω-6_ω-3_ratio, ratio of ω-6 fatty acids to ω-3 fatty acids; pufa_mufa_ratio, ratio of PUFA to MUFA; metab, metabolite; PUFA, polyunsaturated fatty acids; MUFA, monounsaturated fatty acids; VLDL, very low–density lipoprotein; HDL, high-density lipoprotein; LDL, low-density lipoprotein

Through mediation analyses we found all the 14 metabolite measures above (total cholesterol in LDL, apolipoprotein A-I, estimated degree of unsaturation, 22:6-docosahexaenoic acid (mmol/l), ω-3 fatty acids, isoleucine, ratio of phospholipids to total lipids in medium LDL, ratio of total cholesterol to total lipids in small LDL, ratio of 22:6 docosahexaenoic acid to total fatty acids, ratio of 18:2 linoleic acid to total fatty acids, ratio of ω-6 fatty acids to total fatty acids, ratio of polyunsaturated fatty acids to total fatty acids, ratio of ω-6 fatty acids to ω-3 fatty acids, and ratio of PUFA to MUFA) mediated between different dietary patterns and GMS (stdβ ranged −0.037 to −0.001; proportion of mediation ranged 4.21% to 66.18%). However, when comparing the different GMS pairs, the mediation effect of only 5 metabolite measures (i.e. apolipoprotein A1 (APOA1), docosahexaenoic acid (DHA), isoleucine, ratio of DHA to total fatty acids, and the ratio of ω-6 to ω-3 fatty acids) maintained consistently. The indirect associations, which were used to indicate the 5 metabolite measures mediated between dietary patterns and GMS, were shown to be negative (proportion of mediation: APOA1 ranged 6.79% to 41.43%, DHA ranged 6.69% to 57.99%, isoleucine ranged 25.29% to 54.32%, ratio of DHA to total fatty acid ranged 9.34% to 42.37%, and the ratio of ω-6 to ω-3 fatty acids ranged 3.36% to 58.98%). Though higher levels of both ω-3 and ω-6 fatty acids associated with higher adherence to dietary patterns and lower odds of an increased GMS score, no evidence for either of them showed a consistent mediation effect; however, the ratio of ω-6 to ω-3 fatty acids showed a mediation effect, with positively associated with dietary patterns but negatively associated with an increased GMS score (Fig. 2 and Additional file 1: Tables S3–S7).

### Longitudinal assessment of metabolite measures for incidence of type 2 diabetes

According to the yearly follow-up within 7 years, 86 incident type 2 diabetes were identified (19 from NGM and 67 from prediabetes). Most of the newly diagnoses occurred in participants with low adherence to dietary patterns (79% for MED, 83% for DASH and 87% for DHD). The risk of type 2 diabetes was observed to be negatively associated with APOA1 in participants of NGM (RR 0.82, 95% CI 0.75, 0.89) and prediabetes (RR 0.60, 95% CI 0.55, 0.65), respectively; in addition, a negative association was observed between DHA and the risk of type 2 diabetes (RR 0.89, 95% CI 0.83, 0.97) in prediabetes participants but not in NGM participants. However, a higher ratio of ω-6 to ω-3 fatty acids was found to increase the risk of type 2 diabetes from prediabetes participants (RR 1.29, 95% CI 1.05, 1.43), while no evidence was found for NGM participants (Table 4).

**Table 4:** Relative risk of type 2 diabetes according to identified metabolite measures

Variables	Relative risk of type 2 diabetes	
	RR	95% CI
**Type 2 diabetes vs. NGM**		
APOA1	0.82	0.75, 0.89
DHA	0.97	0.79, 1.15
Isoleucine	1.12	0.81, 1.57
Ratio of DHA to total fatty acids	1.01	0.93, 1.09
Ratio of ω-6 to ω-3	1.21	0.94, 1.56
**Type 2 diabetes vs. prediabetes**		
APOA1	0.60	0.55, 0.65
DHA	0.89	0.83, 0.97
Isoleucine	1.16	0.93, 1.47
Ratio of DHA to total fatty acids	0.94	0.82, 1.06
Ratio of ω-6 to ω-3	1.29	1.05, 1.43

The intervals of glucose metabolism statuses were defined as follows: NGM, fasting plasma glucose <6.1 mmol/L; prediabetes, fasting plasma glucose of 6.1–6.9 mmol/L and no hypoglycaemic medications; type 2 diabetes, fasting plasma glucose ≥7.0 mmol/L or hypoglycaemic medications

The adjustments included age (years, continuous), sex (male or female), BMI (kg/m^2^, continuous), level of education (low, middle or high), level of household income (<2000 euros/month, 2000–3750 euros/month, or ≥3750 euros/month), smoking status (never, current or former smoker), daily energy intake (kcal/d, continuous), daily glucose intake (mmol/mol, continuous), estimated glomerular filtration rate (eGFR, ml/min, continuous), total physical activity (h/week, continuous), usage of lipid-modification medication (no or yes), history of cardiovascular disease (no or yes), and the year for metabolomics measurement (2014 or 2016)

Reference group was tertile 1

*P*<0.05 was considered statistically significant

*Abbreviations*: *NGM* normal glucose metabolism, *RR* relative risk, *BMI* body mass index, *kcal* kilocalories, *MED* Mediterranean Diet, *DASH* Dietary Approaches to Stop Hypertension diet, *DHD* Dutch Healthy Diet, *CI* confidence interval, *APOA1* apolipoprotein A-I (g/l), *DHA, 22:6* docosahexaenoic acid (mmol/l)

### Sensitivity analysis

The identified 5 metabolite measures were included in a series of sensitivity analyses, where the results remained consistent after excluding participants with incomplete data on covariates and newly type 2 diabetes diagnoses at baseline (Additional file 1: Tables S8–S12).

## Discussion

Leveraging data from a population-based cohort, this study identified a stable metabolic signature, consisting of 5 metabolite measures (i.e. APOA1, DHA, isoleucine, ratio of DHA to total fatty acids and ratio of ω-6 to ω-3 fatty acids) that consistently mediates the associations between three healthy dietary patterns (i.e. MED, DASH, and DHD) and GMS, indicating the potential of a holistic dietary profile rather than individual foods/nutrients, in regulating circulating metabolites and thereby modifying the glucose metabolism. The current study is one of the largest cohorts that used targeted metabolomic technique (NMR), instead of the widely used untargeted and non-quantified techniques, for blood metabolome investigation. Although it is known that targeted techniques do not achieve global coverage, this technique is thought to be more sensitive, accurate, and specific than the non-targeted metabolomics approach.

Although some healthy dietary patterns have been reported and recommended, the common critical metabolites that suit for multiple dietary patterns and in relation to metabolic disorders have yet to be well investigated and understood. In the current study, the identified metabolites, that showed to be stable in response to nutritional changes, have the potential to identify the early onset of type 2 diabetes, thereby providing a new understanding of the biological mechanisms explaining the association between diet and glucose metabolism. This could directly facilitate preventive nutritional recommendation for type 2 diabetes.

Previous studies already identified individual metabolites (e.g. caffeine, carotenoids, flavonoids, and alkyl resorcinol) being associated with dietary patterns (e.g. Healthy Diet Indicator score) and food intakes (e.g. coffee, vegetables and fruits), indicating that metabolites in biospecimens may serve as biomarkers for assessing diet [34–41]; however, the mediation role of metabolites in the relation between diet and human health is still poorly understood, such as glucose metabolism that was reported to be highly affected by circulating metabolites and diet [42]. Through rigorous analyses, this study firstly demonstrated the participants who adhered to healthier dietary patterns with better metabolically in glycaemic traits (i.e. glucose, HbA1c and HOMA-IR). Although mechanisms driving these dietary pattern-glycaemic traits associations are complex and multi-factorial, the metabolomic profile identified in the current study, i.e. APOA1, DHA, isoleucine, ratio of DHA to total fatty acids and ratio of ω-6 to ω-3 fatty acids, may help to imply the potential pathways for revealing the effects of dietary behaviours on glucose metabolism, which, investigations are therefore needed to elucidate the interplay between specific diet related metabolites and their metabolic and biological pathways.

APOA1, a protein with multiple therapeutic functions, has been reported to be associated with an improved glycaemic control in patients with type 2 diabetes and a decelerated progression of prediabetes to type 2 diabetes [43–45]. Cell and animal studies have confirmed that APOA1 activates protein kinase A (PKA), which trans-locates the transcription factor FoxO1 from the nucleus to the cytoplasm in a process that derepresses transcription of the insulin gene and thereby increases insulin synthesis in β-cells [46]. In-vitro studies have further established that high-density lipoproteins and APOA1 conserve β-cell function by inhibiting apoptosis in a process that is driven by oxysterol efflux and activation of the hedgehog signalling receptor [47, 48]. In addition, there is also mounting evidence that APOA1 improves glycaemic control by increasing glucose uptake into the skeletal muscle and the heart [49]. These observations are supported, at least in part, by findings from the current study which indicated that an increment of plasma APOA1 levels is an increased GMS score, HbA1c level, and HOMA-IR level.

Isoleucine, one of the branched-chain amino acids (BCAA), which mainly derives from food abundant in proteins (e.g. dairy products and meat) and is highly related to metabolic disorders [50–52], was found to be a mediator in the relation between the high adherence to the dietary patterns (i.e. MED, DASH and DHD) and a lower risk of an increased GMS score. Similar findings were observed in a previous study conducted in the USA showing that higher dietary BCAA (i.e. isoleucine, leucine, valine) and plasma BCAA concentrations were jointly associated with an increased risk of incident type 2 diabetes risk [53]. The current study additionally shows that isoleucine mediates 25.29% to 54.32% of the relations between the dietary patterns with (a) NGM vs. type 2 diabetes and (b) prediabetes vs. type 2 diabetes, thereby, suggesting indicating a distinct pathway of diet affecting type 2 diabetes through isoleucine. These findings were strengthened by experimental studies, showing that an increased level of BCAA affects insulin resistance by either activation of the mammalian target of rapamycin complex 1 (mTORC1), which results in uncoupling of insulin signalling at an early stage, or by an impaired BCAA metabolism, thereby causing accumulation of BCAA and mitochondrial dysfunction, which is associated with stress kinase activation and β-cell apoptosis [54]. Therefore, the current study not only provided evidence for a possible role of BCAA (i.e. isoleucine) as a biomarker for type 2 diabetes, but also indicates a plausible mediation role for BCAA between dietary patterns and type 2 diabetes. However, considering the complicated interactions between BCAA and many other metabolites [55], the independent effect of isoleucine on type 2 diabetes should be interpreted with caution and further investigated in future research.

Plasma DHA and the ratio of DHA to total fatty acids were shown to be positively associated with the dietary patterns (i.e. MED, DASH and DHD) and negatively associated with type 2 diabetes. This is in line with a clinical trial conducted in Australia, suggesting that fish oil enriched with DHA reduces insulin resistance and thereby helps to prevent type 2 diabetes [56]. Emerging evidence shows that marine n-3 polyunsaturated fatty acids (PUFAs), eicosapentaenoic acid (EPA), and DHA, are able to ameliorate insulin resistance in rodents, probably via regulating adipocytokines secretion [57–59], inhibiting adipose remodelling [60], lowering inflammation [61], and enhancing mitochondrial function and β-oxidation [62]. A recent study investigated the individual effect of DHA supplementation on glucose metabolism and found that DHA significantly attenuated hyperglycaemia and insulin resistance in db/db mice, which sheds light into the gut-organs axis as a promising target for restoring glucose homeostasis and suggests a therapeutic effect of DHA for treating diabetes [63].

Another interesting finding of the current study is that the ratio of ω-6 to ω-3 fatty acids showed to be positively associated with GMS (overall increment and each pair) and negatively associated with increased scores of all assessed dietary patterns. However, no evidence of either ω-3 and ω-6 fatty acids in mediating dietary patterns and GMS was found. ω-3 and ω-6 fatty acids compete in certain metabolic pathways, e.g. sharing the same conversion enzyme and having inverse biological availability and activity in tissues. Therefore, the combined effect on glucose metabolism has gained wide discussion, but so far remains controversial [64]. Results of the present study are in line with a recently conducted study showing that the ratio of ω-6 to ω-3 fatty acids has the potential to serve as an essential predictive biomarker in the management of patients with type 2 diabetes [65]. In addition, previous research showed that differential intakes of dietary fat subtypes may affect diabetes risk by modifying the phospholipid composition of cell membranes [66]. This effect may play a role in blood glucose regulation through action on insulin secretion and insulin receptor properties [67, 68], which leads to developing normoglycemic blood glucose levels in individuals with the lowest ratio of ω-6 to ω-3 fatty acids. However, the exact balance and mechanism of the ratio of ω-6 to ω-3 fatty acids has not yet been well understood; further research is, therefore, warranted.

Furthermore, by using the longitudinal data on GMS, we observed there was 67 type 2 diabetes developed from 514 prediabetes (13%), compared to 0.97% (19 type 2 diabetes out of 1960 NGM, which indicated the prediabetes with a higher risk to develop to type 2 diabetes. Except for ratio of DHA to total fatty acids, though might be due to the insufficient statistical power, the rest of four identified metabolite measures showed baseline levels longitudinally associated with the risk of type 2 diabetes, which strengthen the robustness of results based on cross-sectional analysis. Particularly the ratio of ω-6 to ω-3 fatty acids with evidence of prospective risk of type 2 diabetes for prediabetes but not NGM, which suggested it may be a key biomarker in predicting and preventing the deterioration of prediabetes. Therefore, metabolic profiling may serve as a more accurate and unbiased method to assess the impact on the relationship between diet and health outcomes.

Main strengths of this study are the large size of this population-based cohort study with oversampling of individuals with type 2 diabetes, which enables accurate comparison of individuals with and without diabetes, and the large number of potential confounders that were considered. Moreover, the use of the NMR platform provided standardized measures of the metabolites, allowing exploration of measures beyond routinely measured biomarkers. However, several limitations should be acknowledged; first, an external replication cohort is warranted to validate the proposed panel of metabolite measures identified in the current study; second, based on an observational study design, any causal inference should be made with caution, since bidirectional associations may exist; third, though the mediation analyses indicate the important roles of metabolites in modulating diet on glucose metabolism, the biological mechanism should be demonstrated with caution given that the identified metabolites might not be involved in the dominant pathways, particularly those with low proportion of mediation effects. fourth, although analyses were adjusted for known potential confounders, the possibility of unmeasured confounding (e.g. the fasting time before blood sample collection, the cooking methods, and the duration and intensity of smoking) and reverse causation remains; fifth, some of the clinical factors and dietary intakes were self-reported, therefore, misclassification errors are likely to have biased these findings toward the null; sixth, in the current study, dietary intakes were assessed by FFQs. Therefore, measurement error and misclassification are unavoidable due to the inability of a FFQ to capture details information (i.e. exact food types), the difficulty in the quantification of the intake, and the high dependency on memory; finally, this sample was restricted to volunteers of European ancestry aged around 60 years at baseline and, therefore, further research is warranted to investigate to what degree these findings can be generalized to other populations.

## Conclusions

In summary, this study suggests that adherence to a healthy dietary pattern (i.e. MED, DASH, or DHD) could affect glucose metabolism status through the regulations of the circulating metabolite levels, particularly through APOA1, DHA, isoleucine, ratio of DHA to total fatty acids and ratio of ω-6 to ω-3 fatty acids. This metabolite signature provides new insights into the understanding of the biological mechanisms of diet on glucose metabolism, which facilitates the use of dietary metabolic profiling for the objective measurement of dietary patterns and for the development of dietary recommendations for a better glycaemic control.

## Supplementary Information

**Additional file 1: Table S1.** Information of included metabolite measures. **Table S2.** Components and scaling methods of dietary patterns used in The Maastricht Study. **Table S3.** Association of metabolite measures with dietary patterns. **Table S4.** Association of metabolite measures with glucose metabolism status, HOMA-IR and HbA1c. **Table S5.** Metabolite measures mediated between MED and GMS. **Table S6.** Metabolite measures mediated between DASH and GMS. **Table S7.** Metabolite measures mediated between DHD and GMS. **Table S8.** Associations of dietary patterns with glucose metabolism status in The Maastricht Study excluding participants with incomplete data on covariates and newly type 2 diabetes diagnoses. **Table S9.** Associations of dietary patterns with HOMA-IR and HbA1c in The Maastricht Study excluding participants with incomplete data on covariates and newly type 2 diabetes diagnoses. **Table S10.** Associations of dietary patterns with identified metabolite measures in The Maastricht Study excluding participants with incomplete data on covariates and newly type 2 diabetes diagnoses. **Table S11.** Associations of dietary patterns with identified metabolite measures in The Maastricht Study excluding participants with incomplete data on covariates and newly type 2 diabetes diagnoses. **Table S12.** Metabolite measures mediated between dietary patterns and GMS in The Maastricht Study excluding participants with incomplete data on covariates and newly type 2 diabetes diagnoses.

## Data Availability

Data are available from The Maastricht Study for any researcher who meets the criteria for access to confidential data; the corresponding author may be contacted to request data.

## Abbreviations

APOA1: Apolipoprotein A1
BCAA: Branched-chain amino acids
BMI: Body mass index
DASH: Dietary Approaches to Stop Hypertension (DASH) Diet
DHA: Docosahexaenoic acid
DHD: Dutch Healthy Diet
EDTA: Ethylenediaminetetraacetic acid
eGFR: Estimated glomerular filtration rate
EPA: Eicosapentaenoic acid
FDR: False discovery rate
FFQ: Food frequency questionnaire
GMS: Glucose metabolism status
HbA1c: Haemoglobin A1c
HOMA-IR: Homeostatic Model Assessment for Insulin Resistance
IR: Insulin resistance
MED: Mediterranean Diet
mTORC1: Mammalian target of rapamycin complex 1
MUFA: Monounsaturated fatty acids
NGM: Normal glucose metabolism
NMR: Nuclear magnetic resonance
OGTT: Oral glucose tolerance test
OR: Odds ratio
PKA: Protein kinase A
PUFA: Polyunsaturated fatty acids
RR: Relative risk
SD: Standard deviation
